# Protective Effect of a Heat‐Stabilized Bacteria Probiotic Preparation (VSL#3‐HS) in a Mouse Model of Ulcerative Colitis Induced by Dextran Sulfate Sodium

**DOI:** 10.1002/mnfr.70594

**Published:** 2026-08-21

**Authors:** Chiara Santucci, Alessio Torcinaro, Francesca De Santa, Maria Cristina Giustiniani, Diego Mora, Stefania Arioli, Lucrezia Laterza, Georgios Strimpakos, Stefano Farioli‐Vecchioli, Carla Petrella

**Affiliations:** ^1^ Institute of Biochemistry and Cell Biology Monterotondo Italy; ^2^ Department of Pathology Fondazione Policlinico Universitario A. Gemelli IRCCS Rome Italy; ^3^ Department of Food Environmental and Nutritional Sciences University of Milan Milan Italy; ^4^ Department of Translational Medicine and Surgery University Cattolica del Sacro Cuore Rome Italy; ^5^ CEMAD Fondazione Policlinico Universitario A. Gemelli IRCCS Rome Italy; ^6^ Institute of Biochemistry and Cell Biology Rome Italy

**Keywords:** antiinflammation, experimental colitis, heat stabilization, nonviable probiotics, recovery of clinical signs

## Abstract

Postbiotics, composed of inanimate microorganisms or their components, are gaining attention for managing intestinal discomfort, being safer than probiotics for vulnerable patients. This study investigates the effects of short‐term consumption of a heat‐stabilized VSL#3 formulation (VSL#3^−^HS) in a mouse model of dextran sulphate sodium (DSS)‐induced colitis. Male C57BL/6 mice were divided into four groups: (1) Placebo (PLA); (2) VSL#3‐HS (1 × 10^9^ cells/mouse/day for 7 days); (3) DSS + PLA (2% DSS in drinking water from days 1–7, PLA from days 4–10); (4) DSS + VSL#3‐HS (2% DSS from days 1–7, VSL#3‐HS from days 4–10). All mice were euthanized on day 11 for tissue collection. VSL#3^−^HS rapidly alleviated clinical signs of the disease, including improvements in disease activity index and colon length. This effect was accompanied by a reduction in colonic pro‐inflammatory mediators (IL‐6, TNF‐α, IL‐1β) and a partial restoration of occludin gene expression in the intestine. However, no improvement was observed in MPO activity or microscopic tissue damage. The experimental findings demonstrate the efficacy of postbiotics in a murine model of UC and support further investigation into the efficacy of the heat‐stabilized VSL#3‐HS formulation to assess its potential applicability in human studies.

## Graphical abstract

Protective effect of heat‐stabilized VSL#3‐HS in DSS‐induced murine colitis. Administration significantly ameliorates clinical signs, improving disease activity index, weight loss, and colon shortening. VSL#3‐HS reduces colonic pro‐inflammatory mediators (IL‐6, TNF‐α, IL‐1β) and upregulates occludin junction gene expression, highlighting its potential human applicability.

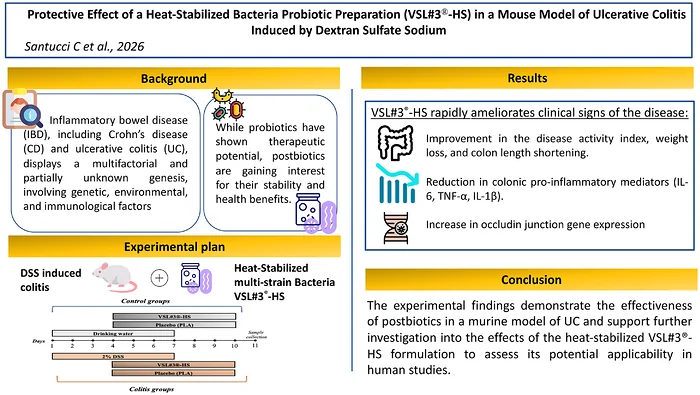

## 1 Introduction

Inflammatory bowel disease (IBD), encompassing Crohn's disease (CD) and ulcerative colitis (UC), is a chronic and relapsing condition with a multifactorial etiology involving genetic, environmental, and immune‐related factors [1, 2, 3]. While CD can affect any segment of the gastrointestinal tract (most commonly the small intestine and the proximal colon), UC is confined to the large intestine and is characterized by continuous mucosal inflammation, disruption of the epithelial barrier, and an increased risk of colorectal cancer [4, 5, 6]. To investigate the pathogenesis of UC and evaluate potential therapeutic approaches, the dextran sulphate sodium (DSS)‐induced colitis mouse model is widely used. This model closely mirrors key clinical and pathological features of human UC, including diarrhea, rectal bleeding, weight loss, mucosal ulceration, colon shortening, and tumor susceptibility [6]. Given its colon‐restricted inflammation and the involvement of dysbiosis in disease progression, the DSS model is well suited for assessing the preventive and therapeutic effects of probiotic and postbiotic interventions [7].

VSL#3 is an extensively studied probiotic mixture comprising eight bacterial strains and a high cell density (450 billions of CFU per sachet) [8, 9]. VSL#3 has been shown to ameliorate DSS‐induced colitis in a murine model by downregulating T follicular helper cells [10] and to prevent the neoplastic progression in a murine model of UC which was accompanied by gut microbiota modulation [11].

Recently, the use of postbiotics to manage intestinal discomforts is receiving great interest in the scientific community [12]. Postbiotics have been defined by the International Scientific Association of Probiotics and Prebiotics (ISAPP) as a “preparation of inanimate microorganisms and/or their components that confers a health benefit on the host” [13]. Effective postbiotics must contain nonviable microbial cells or cell components, with or without metabolites, that contribute to the observed health benefits [13]. Such inanimate microorganisms possess promising health effects, as they contain various physiologically active components.

The growing interest in postbiotics is justified by their potential to overcome several limitations associated with live probiotics. Unlike probiotics, postbiotics are more stable, less affected by environmental or gastrointestinal conditions, and easier to standardize and incorporate into food, beverages, and pharmaceutical formulations [14].

Moreover, clinical evidence indicates that the administration of live microorganisms in patients with critical conditions, such as immunocompromised individuals, may pose safety concerns [15, 16, 17]. The choice of inanimation method is crucial, as the health benefits may vary depending on the conditions employed for inactivation. Recent research investigated the effect of heat‐inactivated probiotics (121°C for 20 min) on pre‐clinical models of colitis, revealing a potential use of dead microorganism in preventing colitis [18]. More recent studies have explored the protective effects of non‐viable multi‐strain preparations in murine models of colitis [19, 20]. To date, only one study exist investigating in rats the beneficial effects of nonviable (heat‐killed) multi‐strain bacterial mixtures derived from the viable VSL#3 preparation in DSS‐induced colitis [21].

In light of the extensively documented beneficial effects of the probiotic mixture VSL#3 in the treatment and prevention of colitis in both preclinical models and clinical trials [22, 23], the present study was designed to investigate a novel heat‐stabilized formulation of VSL#3 compound (hereafter referred to as VSL#3^−^HS), produced by pasteurization, with the aim of evaluating its efficacy in a murine model of DSS‐induced colitis. We predict that short‐term administration of VSL#3^−^HS after induction of colitis rapidly ameliorates clinical signs of the pathology, accompanied by decrease of colonic inflammatory indices (IL‐6, TNF‐α, IL‐1β) and by a partial recovery of the intestinal tight junction integrity.

## 2 Materials and Methods

### 2.1 Animals

Male C57BL/6 mice were purchased from Charles River Laboratories and were allowed to adapt to their environment until they were 8 weeks old. Mice were housed under a continuous 12 h light/12 h dark cycle at a constant temperature of 21°C, with freshly water and food ad libitum. Housing, experimental protocols and procedures were conducted in accordance with the European Union Directive of September 22, 2010 (2010/63/EU), following guidelines of the institutional Animal Research Ethical Committee at CNR‐IBBC and approved by the Italian Ministry of Health (Legislative Decree Number 549/2020‐PR).

### 2.2 Nonviable Probiotic Preparation and Evaluation of the Microbiological Quality

The investigational product, VSL#3‐HS, was a mixture containing eight heat‐stabilized, freeze‐dried bacterial strains: *Bifidobacterium animalis* subsp. *lactis* BL03, *B. animalis* subsp. *lactis* BI04, *B. breve* BB02*, Lactobacillus acidophilus* BA05, *Lactobacillus helveticus* BD08, *Lacticaseibacillus paracasei* BP07, *Lactobacillus plantarum* BP06, and *Streptococcus thermophilus* BT01.

VSL#3‐HS was kindly donated by CSL company (CSL Srl, Zelo Buonpersico, LO, Italy). More details on nonviable probiotic preparation have been included in Supporting Information.

VSL#3‐HS (Batch NTL3999 exp 12/2024 – dosage 100 BLN/stick, provided by Beingpharma Srl) also contained maltose as an excipient. During the study, samples were prepared in order to provide 1 × 10^9^ heat‐stabilized cells/mouse/day based on the cell density quantification obtained by flow cytometry. Placebo contained maltose only. Solutions were formulated to ensure both groups received an identical maltose dosage per mouse.

The detailed description of the methods analyzing the microbiological quality of VSL#3‐HS (total cells quantification, viability evaluation, and microbial composition) has been reported in Supporting Information.

The VSL#3‐HS and placebo were packed in equal sachets and were indistinguishable based on organoleptic properties.

### 2.3 Experimental Protocol and Samples Collection

Male C57BL/6 mice were randomly assigned to the following experimental groups:

1. Controls: Mice receiving standard drinking water from day 1 to day 7.
2. Colitis: Mice receiving 2% DSS in drinking water from day 1 to day 7.

**FIGURE 1:**
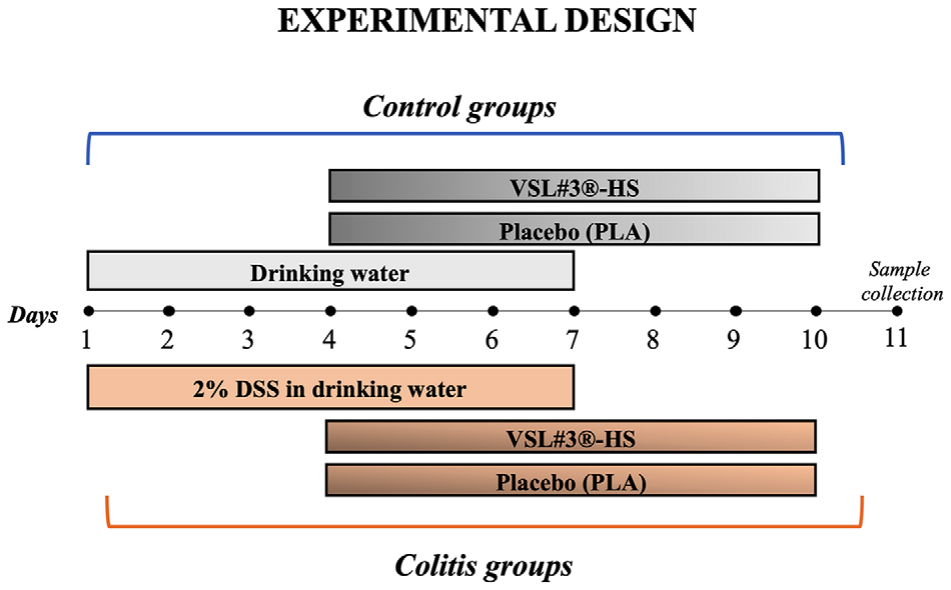
Schematic representation of the experimental protocol. From day 1 to 7, the control group received drinking water, while the colitis group received drinking water supplemented with 2% DSS. From day 4 to 10, both groups received either placebo (PLA) or VSL#3‐HS via oral gavage.

From day 4 to day 10, both Control and Colitis mice were administered either a placebo (PLA; *n* = 5 and *n* = 8, respectively) or VSL#3‐HS (1 × 10^9^ cells/mouse/day; *n* = 5 and *n* = 10, respectively) via oral gavage. Experimental plan has been outlined in Figure 1.

To induce acute colitis, DSS (MW: 36 000–50,000) (MP Biomedicals, Cat#160110) was dissolved in drinking water at concentration of 2% DSS and given to mice from Day 1 to Day 7. Treatment with Placebo (PLA) and VSL#3‐HS was administered via gavage from Day 4 to Day 10. All mice were euthanized on Day 11.

### 2.4 Disease Activity Index Score

Macroscopic parameters (weight loss, stool consistency, and rectal bleeding) were daily registered for the Disease Activity Index (DAI) in all experimental groups. The scores, defined as in Table 1, were calculated to evaluate the clinical progression of colitis. Moreover, daily DSS solution consumption by the mice was recorded in order to evaluate potential difference with tap water intake.

**TABLE 1:** Definition of scores assigned to DAI parameters.

Weight loss	Stool consistency	Bleeding
0 = no loss	0 = normal	0 = no blood
1 = 1%–5%	1 = soft	2 = visible blood in feces
2 = 5%–10%	2 = very soft	4 = blood around anus and bleeding
3 = 10%–20%	3 = loose	
4 = >20%	4 = diarrhea	

### 2.5 Tissue Collection

After the sacrifice, colon samples were isolated, and the length from the anus to the ileocecal junction was measured and registered, to determine colon length changes under different treatments. Starting from the distal colon (adjacent to the rectum, excluded from the sampling) and moving toward the cecum, segments were collected for: (1) RNA extraction, (2) protein extraction, (3) MPO activity assay, and (4) histological analysis. All samples were stored at −80°C.

### 2.6 RNA Extraction and qRT‐PCR

Total RNA was obtained by homogenizing colon samples with a tissue homogenizer (Tissue Ruptor Qiagen Cat #: 990890) in TriReagent (Sigma–Aldrich Cat#: T9424). The RNA extraction was performed following TriReagent manufacturer's protocol. Then RNA extracts were purified via lithium chloride method to remove DSS traces (Viennois et al., 2018). RNA was quantified with NanoDrop (Thermo Scientific NanoDrop 2000C). For mRNA analysis, 500 ng of RNA was retrotranscribed with random primers and RT kit (Thermo Fisher Cat#: 8080234). qPCR analysis was performed with SYBR Green Master Mix (Eppendorf, Primer Design Cat#: T PRECPLUS‐R‐SY) and using primer pairs manually designed with Primer3 (primer3.ut.ee). The reactions were run on 7900HTABI prism PCR machine (Applied Biosystems). All the murine expression primers used in this study span an exon‐exon junction. The sequences of murine expression oligonucleotides for qPCR are listed in Table 2. All values were obtained in duplicate or triplicate, and the analysis of output values was made using standard ΔΔCt method.

**TABLE 2:** Murine expression primers.

Gene symbol	Forward	Reverse
GAPDH	CACCATCTTCCAGGAGCGAG	CCTTCTCCATGGTGGTGAAGAC
IL‐6	TCCTCTCTGCAAGAGACTTCC	TTGTGAAGTAGGGAAGGCCG
IL‐1β	GACCTTCCAGGATGAGGACA	TCCATTGAGGTGGAGAGCTT
TNF‐α	TCTTCTCATTCCTGCTTGTGG	CACTTGGTGGTTTGCTACGA
ZO‐1	GAGTTTCGGGTCCGAGGAG	AAATCCAAACCCAGGAGCCC
OCLN	ACAGTCCAATGGCCTACTCC	TGCCTGAAGTCATCCACACT

### 2.7 Cryosections, Histology and Immunostaining

Colonic portions were embedded in OCT (TissueTek) and frozen in liquid nitrogen‐cooled isopentane (Sigma‐Aldrich). Embedded colon samples were then divided into 8 µm sections on a Leica cryostat (Leica CM1850UV).

For Hematoxylin‐Eosin staining, colon sections were fixed with 4% paraformaldehyde (BDH) and then stained with hematoxylin and eosin (Hematoxilyn: Sigma–Aldrich Cat#: HHS32; Eosin: Sigma–Aldrich Cat#: HT110332) dyes, followed by sequential dehydratation in 75%, 90%, 100% ethanol (Sigma–Aldrich) and xylene (Sigma–Aldrich) and mounted with EUKITT (Sigma–Aldrich Cat#: 03989). Images for histological analysis were acquired using a motorized Leica LMD7000 microscope mounting a DFC310FX cam using LAS X software (Leica) with a 20X objective. The severity of induced colonic inflammation was graded morphologically using a semiquantitative scale by the same histopathologist in a single‐blind fashion. We employed the histological scoring system previously described by Rachmilewitz for grading the severity of DSS‐induced colitis in rodents [24]. This grading system considers the following 5 structural parameters scored on a scale of 0 to 4: depth and extent of the ulcer, presence of inflammation, extent of inflammation, and location of fibrosis (Table 3).

**TABLE 3:** Criteria for scoring the histological lesions.

Score	Depth of the ulcer	Extent of the ulcer	Presence of inflammation	Extent of inflammation	Location of fibrosis
**0**	Absence of ulcer	Absence of ulcer	Absence of inflammation	Absence of inflammation	Absence of fibrosis
**1**	Mucosal involvement	Punctate	Minimal	Mucosal	Mucosa only
**2**	Mucosal and submucosal involvement	Minimal	Mild	Mucosal/submucosal involvement	Mucosa/submucosa
**3**	Penetration of muscularis propria	Moderate	Moderate	Mucosal/submucosal and muscle involvement	Including muscle layer
**4**	Penetration of muscularis propria	Widespread	Severe	Full thickness involvement	Full thickness fibrosis

For immunofluorescence, colon sections were fixed with 4% paraformaldehyde. After blocking with 4% BSA (Jackson Lab, 105696) in PBS + GS 3% + 0.3% Triton, colonic slides were incubated overnight with primary antibodies, at indicated dilutions, at 4°C. Samples were then washed with 1% IgG‐free BSA and incubated with an appropriate secondary antibody for 1 h, at room temperature. The nuclei were counterstained with 4’,6 diamidino‐2‐phenylindole (DAPI; ThermoFisher Cat#: D1306) and then rinsed in PBS three times. Sections were mounted in glycerol 80% (AppliChem). Colonic cryosections were stained with the following primary antibodies at indicated dilutions: 1:200 Anti‐ZO1, mouse (Invitrogen, Cat#:33‐9100); 1:500 Anti‐Occludin, rabbit (Proteintech, Cat#13409‐1‐AP). Secondary antibodies: Alexa Fluor 488 Donkey anti‐mouse (H+L) (Jackson ImmunoResearch #715‐545‐150); Alexa Fluor Cy3 Donkey anti‐rabbit (H+L) (Jackson ImmunoResearch #711‐165‐152). Immunostainings were visualized by a confocal laser scanning microscope (Olympus FV1200) with 40X magnification. Pinhole size, image resolution, color depth, and scan speed were the same for all the samples analyzed. Immunostainings were visualized using a confocal laser scanning microscope (Olympus FV1200) at 40× magnification. Pinhole size, image resolution, color depth, and scan speed were kept constant across all analyzed samples. Occludin and ZO‐1 immunofluorescence were quantified using Fiji (ImageJ) by converting images to 8‐bit format and then measuring the mean gray value of the signal across the entire image area, using “Measure” function. For each sample, three fields per section were analyzed, across at least three sections.

### 2.8 MPO Activity Assay

MPO activity assay was performed on colonic samples according to manufacturing protocol of Myeloperoxidase Colorimetric Activity Assay Kit (Sigma–Aldrich, Cat#MAK068). Briefly, colonic samples were rapidly homogenized in 4 volumes of MPO Assay Buffer and centrifuged at 13 000 x g for 10 min at 4°C to remove insoluble materials. All unknown samples and standards were run in duplicates. Absorbance was measured at 412 nm, using VarioSkan (ThermoScientific). 1 µl of each sample was normalized using protein quantification by the Bradford method.

### 2.9 ELISA Assay

Colonic samples were homogenate with a tissue homogenizer (Tissue Ruptor Qiagen, Cat #: 990890) in PBS 1X and ELISA assay for cytokines IL‐6 (Mouse IL‐6 ELISA Kit – 96T, Cohesion Biosciences, CEK1785), TNF‐α (Mouse TNF alpha ELISA Kit – 96T, Cohesion Biosciences, CEK1565), IL‐1β (Mouse IL‐1ß ELISA Kit – 96T, Cohesion Biosciences, CEK1788) was performed according to manufacturing protocol.

### 2.10 Statistical Analysis

Statistical analysis was carried out with GraphPad Prism (San Diego, CA, USA). Data were presented as mean ± S.E.M. For comparison of groups, parametric data were analyzed using a two‐way ANOVA or one‐way ANOVA where appropriate. Post‐hoc comparisons within logical sets of means were performed using the Tukey's test, the use of which is permissible or even recommended also in the absence of significant main or interaction effects in the ANOVA, to minimize frequency errors of both type I and II following the indications given by Wilcox [25]. For all analyses, *p* < 0.05 was considered significant.

## 3 Results

### 3.1 Consumption of VSL#3‐HS Ameliorated Macroscopic Parameters of Colitis

DSS was administered to male C57BL/6 mice to induce acute colitis and then the effect of VSL#3‐HS was evaluated in vivo and ex‐vivo; a schematic representation of experimental protocol and experimental groups is reported in Figure 1.

Daily solution consumption reported in Figure S1 (see Supporting Information) showed no differences among the groups (PLA, DSS‐PLA, DSS‐ VSL#3‐HS), clearly demonstrating that any kind of treatment could influence the intake.

**FIGURE 2:**
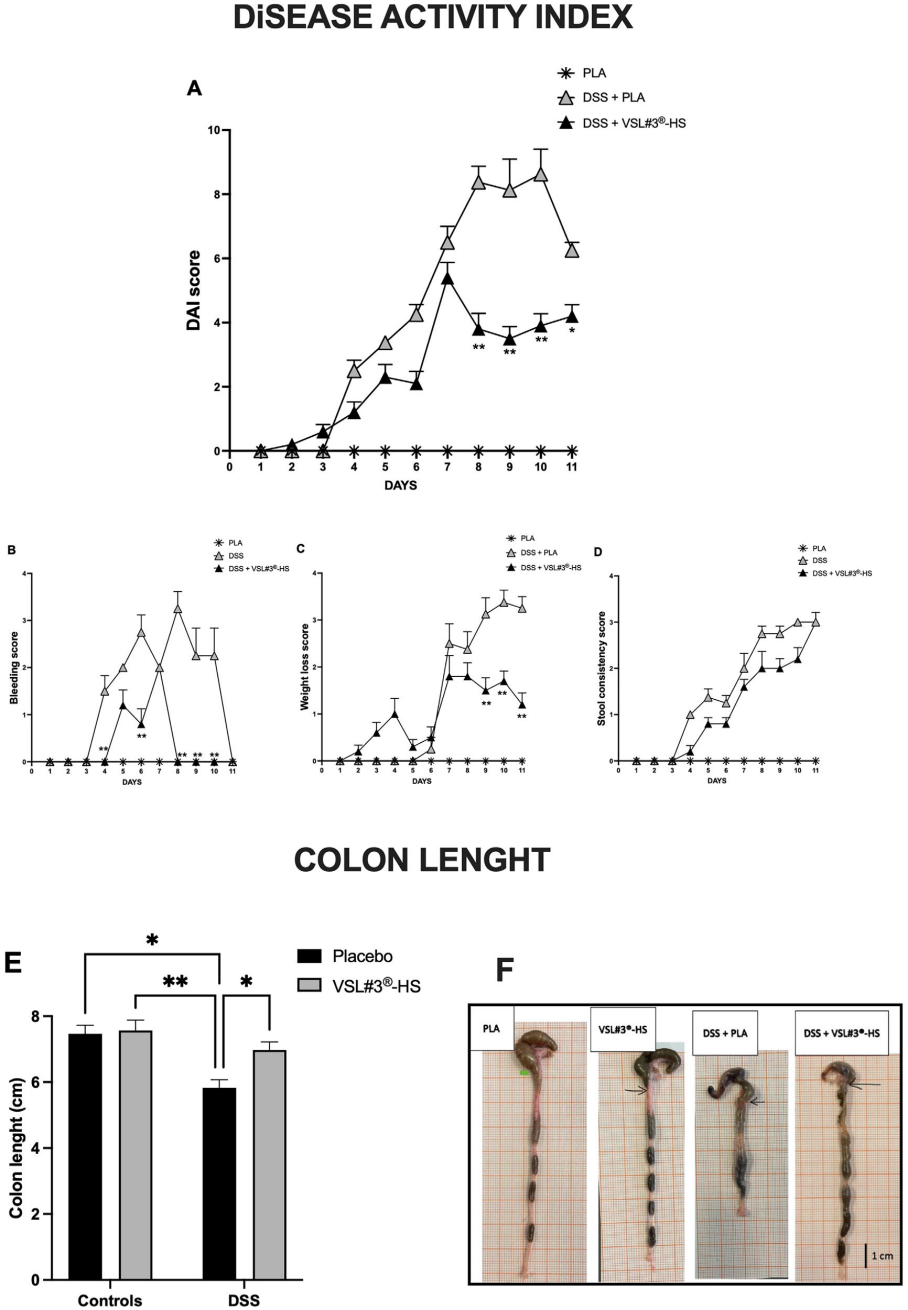
Macroscopic parameters included in the study. Panel A illustrates the Disease Activity Index in PLA, DSS + PLA and DSS + VSL#3‐HS groups, during experimental time, calculated by combining the measured scores of rectal bleeding, weight loss and stool consistency. Panel B, C and D show separately the bleeding score, the weight loss score, and the stool consistency score, respectively. Data were presented as mean ± S.E.M.; PLA = n.5, DSS = n.8, DSS + VSL#3‐HS = n.10: two‐way ANOVA followed by Tukey's multiple comparison, *p < 0.05; **p < 0.01. Asterisks indicate statistical significance between DSS+ PLA versus DSS + VSL#3‐HS groups. Graphic (panel E) and representative images (panel F) showing colon length assessment in different experimental groups. Data were presented as mean ± S.E.M.; PLA = n.5, VSL#3‐HS = n.5, DSS = n. 8, DSS + VSL#3‐HS = n.10: two‐way ANOVA followed by Tukey's multiple comparison, *p < 0.05; **p < 0.01.

Figure 2 illustrates the main macroscopic parameters put in consideration in the study.

DAI score (panel A), considering body weight loss, stool consistency and rectal bleeding, showed that consumption of VSL#3‐HS was able to favor the recovery from the indexes of pathology increased upon colitis induction. Interestingly, separate analysis of the individual components comprising the DAI score (panels B, C and D) revealed that VSL#3‐HS treatment significantly reduced rectal bleeding and mitigated weight loss. In contrast, stool consistency, which was impaired in colitic mice, remained unaltered. Since no significant differences were observed between the VSL#3‐HS and PLA groups, these data were not included in the DAI panels (A, B, C and D).

Colon length (panel E and F) was significantly reduced in DSS mice [F_(1,18) = _11.68; p = 0.0031], whilst VSL#3‐HS restored the physiological condition [F_(1,18) = _8.381: p = 0.0097].

Collectively, these parameters demonstrate that the short‐term administration of VSL#3‐HS can rescue the macroscopic pathological hallmarks of DSS‐induced colitis.

### 3.2 VSL#3‐HS Did Not Modulate Histological Alteration and Colonic Inflammation Upon Colitis Induction

We performed the histological analysis upon DSS‐induced colitis by hematoxylin/eosin staining on colon cryosections, followed by an evaluation of the Rachmilewitz score, to evaluate at morphological level the severity of colitis and the effect of VSL#3‐HS at this stage.

**FIGURE 3:**
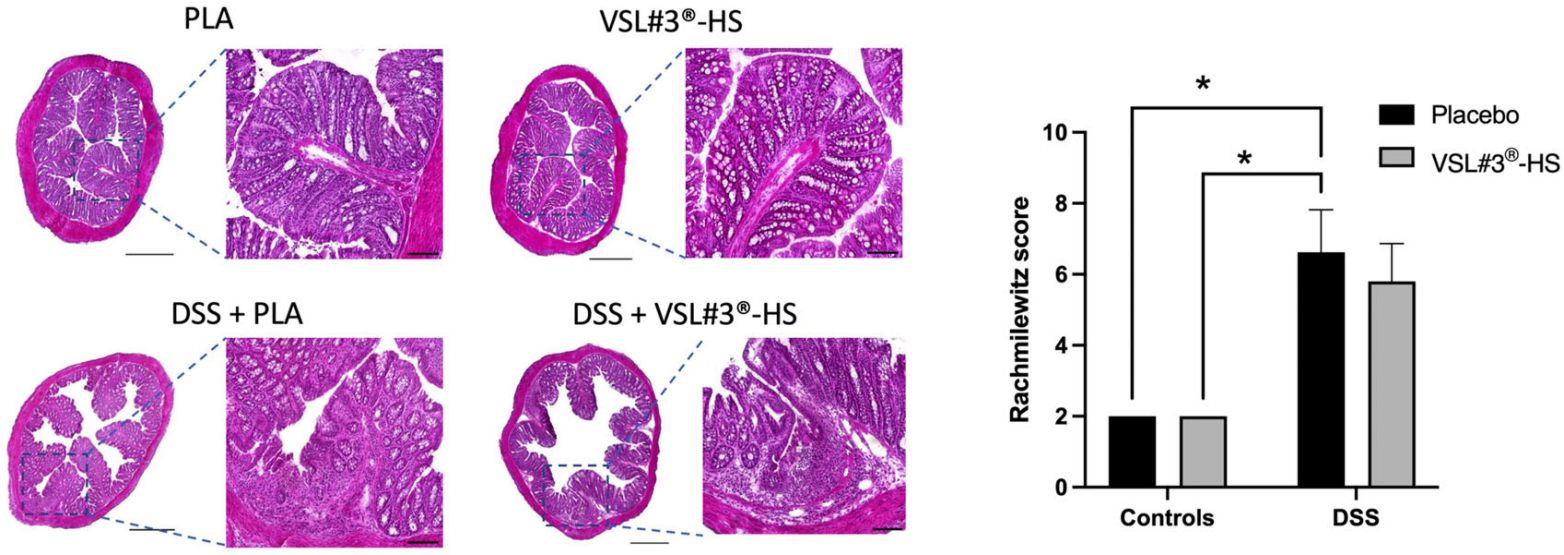
Colonic histological score. Representative images of Hematoxylin‐Eosin staining from different experimental groups (PLA, VSL#3^−^HS, DSS+PLA and DSS+VSL#3‐HS). Histograms represent the Rachmilewitz score. Data were presented as mean ± S.E.M.; PLA = n.3, VSL#3‐HS = 3 DSS = n. 7, DSS + VSL#3‐HS = n.6: two‐way ANOVA followed by Tukey's multiple comparison, *p < 0.05; **p < 0.01. Scale bar: 500 µm for the uncropped pictures, 100 µm for zoomed images.

As shown in Figure 3, DSS treatment induced a significant increase in the Rachmilewitz score [F_(1,15)_ = 6.800: p = 0.0198]. One‐week treatment during colitis with VSL#3‐HS did not modify histological alterations.

### 3.3 Consumption of VSL#3‐HS Did Not Restore MPO Activity, but Decreased Colonic Cytokine Levels Upon Colitis Induction

**FIGURE 4:**
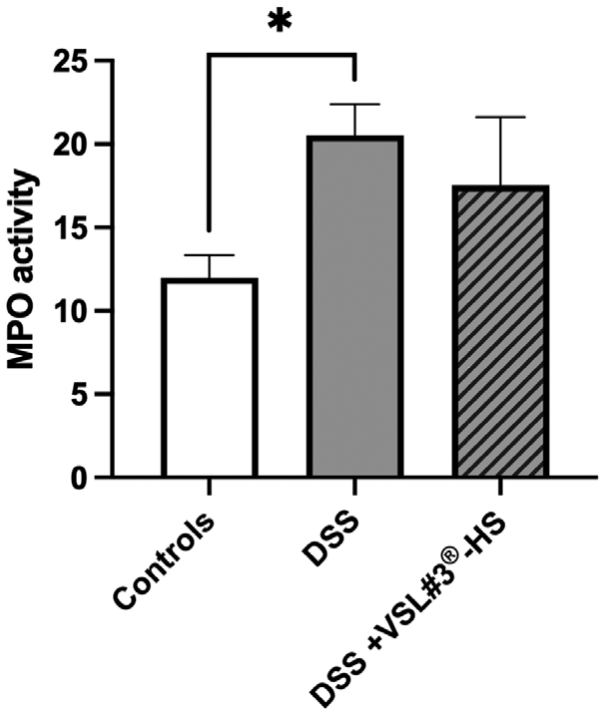
Colonic MPO activity. Histograms represent the MPO activity for each experimental group (PLA, DSS+PLA and DSS+VSL#3‐HS). Data were presented as mean ± S.E.M.; PLA = n.3, DSS = n.3, DSS + VSL#3‐HS = n.4: one‐way ANOVA followed by Tukey's multiple comparison, *p < 0.05.

Inflammatory parameters associated with colitis have been evaluated. MPO activity is an accepted biomarker of neutrophil invasion in the gut. Indeed, the MPO activity assay was performed on colonic samples derived from mice upon DSS‐induced colitis and VSL#3‐HS administration. As shown in Figure 4, ANOVA analysis revealed a “disease” effect, as expected, causing an increase of colonic MPO activity in DSS‐mice [F_(2,7) = _4.708; p = 0.041], but VSL#3‐HS treatment did not restore control condition.

Colonic inflammation is a well‐established hallmark of DSS‐induced colitis and represents a key therapeutic target to be mitigated during intervention strategies.

**FIGURE 5:**
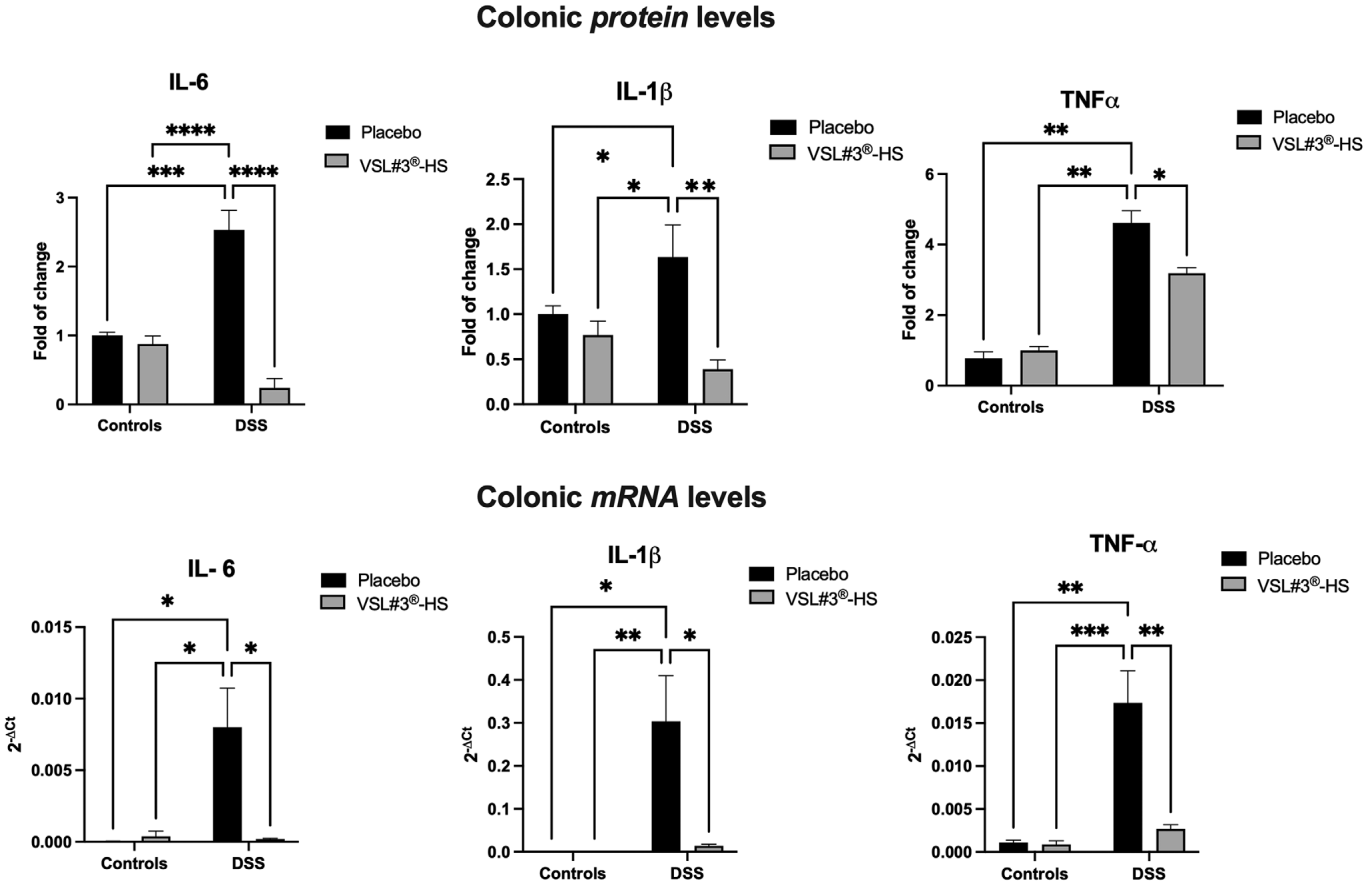
Colonic mRNA and protein levels of pro‐inflammatory cytokines. Histograms represent IL‐6, IL‐1β and TNF‐α levels for each experimental group. Data were presented as mean ± S.E.M.; PLA = n.5, VSL#3‐HS = n.5, DSS = n.8, DSS + VSL#3‐HS = n.10: two‐way ANOVA followed by Tukey's multiple comparison, *p < 0.05; **p < 0.01; ***p < 0.001.

As shown in Figure 5, DSS induced a significant increase of IL‐6, IL‐1β and TNF‐α both at protein (IL‐6: [F_(1,22) _= 5.198: p = 0.0327]; IL‐1β: [F_(1,22) _= 6.102: p = 0.031]; TNF‐α [F_(1,21) = _84.32: p < 0.001] revealed by ELISA assay (Figure 5A–C) and at gene regulation level (IL‐6: [F_(1,24) = _4.695: p = 0.007]; IL‐1β: [F_(1,24) = _5.435: p = 0.0285]; TNF‐α [F_(1,23) = _12.76: p = 0.001], as disclosed by qRT‐PCR (Figure 5D–F).

Collectively, VSL#3‐HS consumption was able to counteract all the inflammatory parameters analyzed, known as crucial mediators of IBD (protein: IL‐6 [F_(1,22) = _38.03: p < 0.001, Figure 5A]; IL‐1β [F_(1,22) = _13.58: p = 0.013, Figure 5B]; TNF‐α [F_(1,21) = _8.015: p < 0.01, Figure 5C]—mRNA IL‐6 [F_(1,24) = _8.470: p = 0.0077, Figure 5D]; IL‐1β [F_(1,24) = _8.456: p = 0.007, Figure 5E]; TNF‐α [F_(1,231) = _14.97: p = 0.0008, Figure 5F].

### 3.4 Consumption of VSL#3‐HS Partially Reestablished Colonic Tight Junctions

The maintenance of the structural integrity of the intestinal barrier is essential for the regulation of intestinal permeability and the preservation of intestinal homeostasis.

To this end, colonic occludin and ZO‐1, two tight junction markers, were evaluated at both the mRNA level and the protein level, the latter assessed by immunofluorescence analysis on colonic cryosections.

**FIGURE 6:**
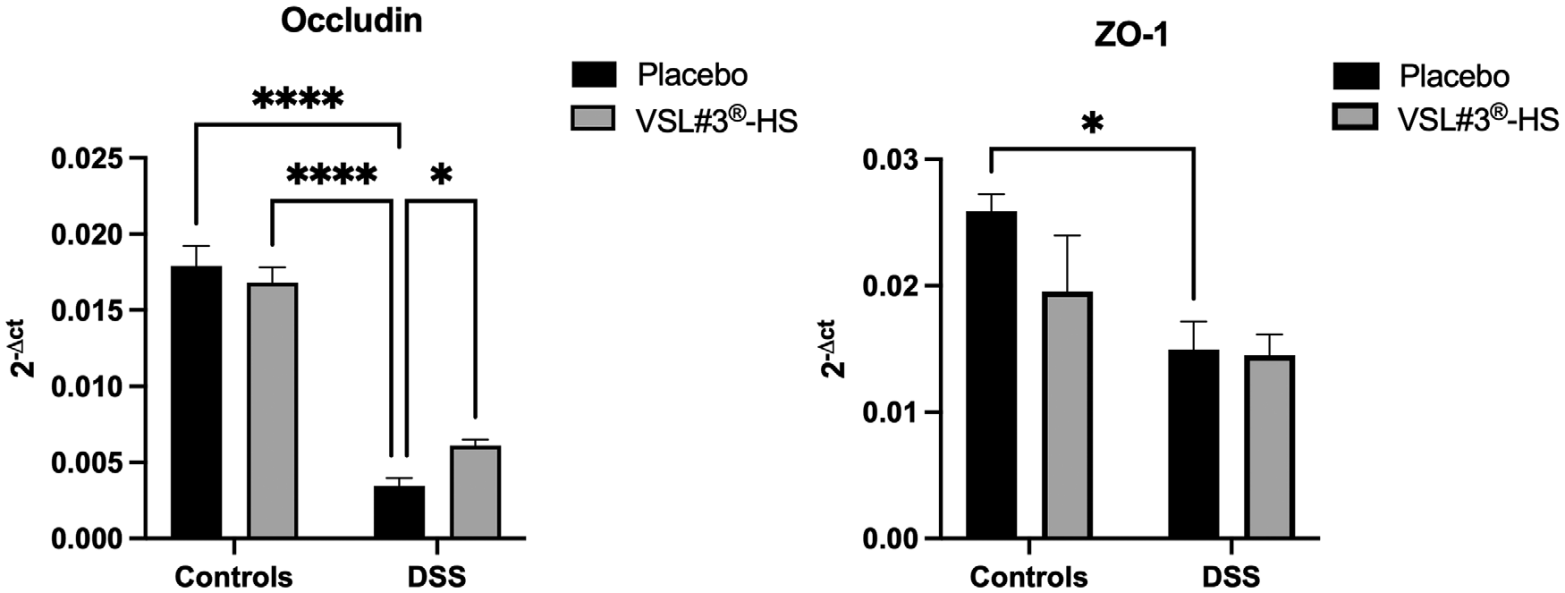
Colonic occludin and ZO‐1 mRNA levels. Histograms represent the mean ± SEM of for each experimental group (PLA = n.5, VSL#3‐HS = n.5, DSS = n.8, DSS + VSL#3‐HS = n.10: two‐way ANOVA followed by Tukey's multiple comparison. *p < 0.05; ****p < 0.001).

As shown in Figure 6, both occludin and ZO‐1 mRNA were downregulated after seven days DSS treatment (occludin: [F_(1,23) = _279.9: p < 0.0001, Figure 6A]; ZO‐1: [F_(1,24) = _10.31: p = 0.0037], Figure 6B), disclosing an impairment in intestinal functionality. Interestingly, VSL#3‐HS treatment partially reestablished occludin decrease F_(1,23) = _25.03: p < 0.042, Figure 6A], without affecting ZO‐1 levels [F_(1,24) = _1.869: p = 0.184, Figure 6B].

**FIGURE 7:**
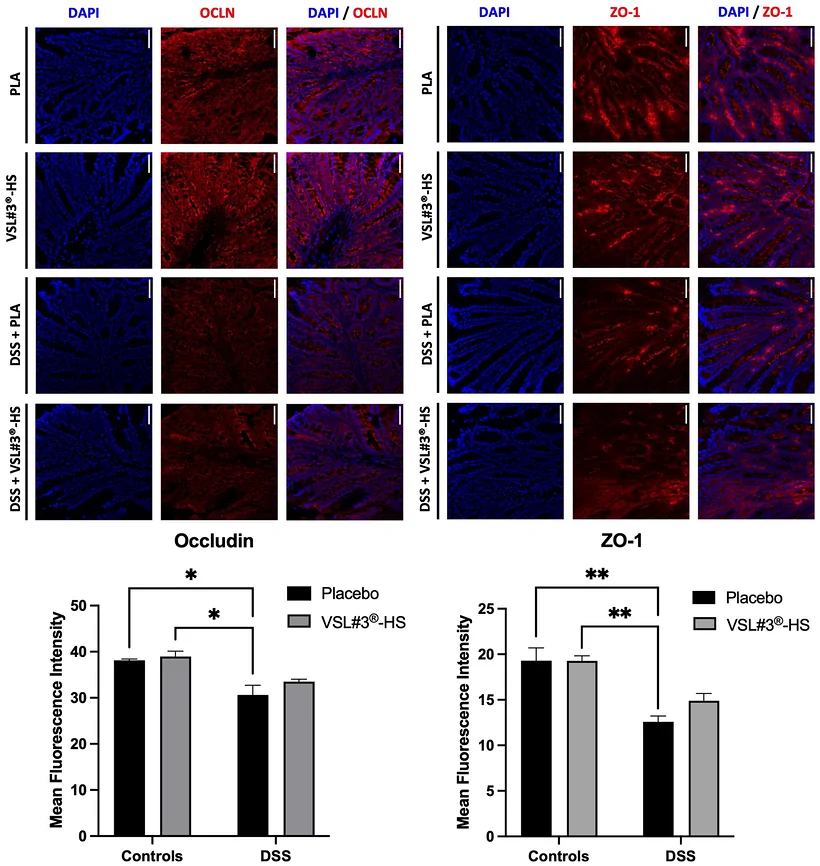
Colonic occludin and ZO‐1 protein expression. Representative imagines of occludin and ZO‐1 immunofluorescence. Histograms represent the mean ± SEM of fluorescence intensity for each experimental group (PLA = n.2, VSL#3‐HS = n.2, DSS = n.3, DSS + VSL#3‐HS = n.5: two‐way ANOVA followed by Tukey's multiple comparison. *p < 0.05; ****p < 0.001). Scale bar: 50 µm.

When we explored the presence and the localization of occludin and ZO‐1 by immunofluorescence (Figure 7A), we confirmed the decrease of occludin [F_(1,7)_ = 22.85, p = 0.002, Figure 7B] and ZO‐1 [F_(1,8)_ = 32.76, p = 0.0004, Figure 7C] junctions in DSS‐treated mice. Immunofluorescence assay did not reveal any changes in occludin [F_(1,7)_ = 1.843, p = 0.216, Figure 7A,B] and ZO‐1 F_(1,8)_ = 1.400, p = 0.270, Figure 7A,C] levels after VSL#3‐HS consumption. These data demonstrate that VSL#3‐HS is able, upon DSS‐induced colitis, to partially counteract the reduction of occludin tight junction, thus contributing to preserve intestinal integrity.

## 4 Discussion

In the present study, we have demonstrated that the short‐term administration (1 week) of a mix of probiotics stabilized by heat (VSL#3‐HS) in mice, upon DSS‐induced colitis, rapidly improved macroscopic disease signs (colon shortening, disease activity index). This effect was accompanied by a potent anti‐inflammatory action at the colonic level, as evidenced by the reduction of inflammatory mediators relevant for IBD (IL‐6, IL‐1β, TNF‐α), at both the mRNA and protein level. Interestingly, DSS‐induced histological alteration and myeloperoxidase activity were not normalized by short‐term postbiotic consumption. Finally, VSL#3‐HS partially preserved intestinal integrity by mitigating the reduction of colonic occludin expression.

IBD pathogenesis involves genetic, environmental, and microbiota‐related factors [26, 27, 28]. Specifically, bacterial invasion by pathogenic microorganisms and changes in protective factors (intestinal barrier breakdown, decreased colonic production of SCFAs) contribute to IBD pathogenesis and lead to intestinal dysbiosis [28, 29, 30]. The use of probiotics in IBD aims to restore gut dysbiosis by rebalancing the intestinal microbiota, supporting mucosal barrier function, and modulating the immune response [31]. Research in this field seeks to leverage eubiosis as a therapeutic strategy.

Several studies have established that probiotic consumption (single‐strain and/or multi‐strain formulations) provides a benefit to the health of the host by modulating the microbiota.

Notably, postbiotics, comprising non‐viable microbial cells [13]. and bioactive metabolites such as SCFAs, can provide health benefits (including anti‐inflammatory effects, oxidative stress sand immune modulation [32, 33]) independently of probiotic viability [34, 35, 36]. A key advantage of using postbiotics, compared to living microorganisms, is the lack of risks such as bacterial translocation and acquisition of antibiotic resistance [37]. As stabilized products, postbiotics offer easier storage (often at room temperature) and can be combined with probiotics or conventional therapies to support long‐term homeostasis.

Several studies conducted with postbiotics, primarily produced by a single non‐viable bacterial strain (mainly *Lactobacillus* and *Bifidobacterium* species), have shown positive effects in vivo, including hypocholesterolemic [32], anxiolytic [38], antidepressive [39], and anti‐obesogenic effects in obese mouse models [40]. Additionally, in vitro studies have revealed the beneficial properties of these single‐strain nonviable preparations, such as immune‐enhancing effects in RAW264.7 cells [41], anticancer effects in AGS cell lines [42], and hepatoprotective effects in human HepG2 cells [43]. Interestingly, pasteurized *Akkermansia muciniphila* has emerged as a highly effective and safe therapeutic agent, often outperforming its live counterpart in treating metabolic and inflammatory disorders. This mucin‐degrading bacterium shows significant promise in clinical and preclinical models for managing obesity, diabetes, and cancer by modulating host immune responses and metabolic pathways [44].

Recent research on the effects of proteins and metabolites secreted by VSL#3 strains (the secretome) on intestinal epithelia suggests that VSL#3 directly influences the intestinal epithelial barrier, enhancing intestinal integrity by promoting cell proliferation and reducing apoptosis [45].

In our experimental study, the consumption of the non‐viable formulation VSL#3^−^HS (produced through heat stabilization of the individual strain followed by mixing to form the final product) rapidly improved the clinical signs of experimental colitis. Specifically, the disease activity index showed significant improvement after just five days of treatment with VSL#3‐HS, compared with DSS‐treated control mice. Interestingly, its greater efficacy in reducing rectal bleeding and mitigating weight loss than in restoring stool consistency supports the hypothesis that the heat‐stabilized compound acts primarily through anti‐inflammatory pathways. Indeed, it is well established that rectal bleeding and weight loss are directly associated with mucosal inflammation, whereas diarrhea is primarily linked to alterations in intestinal motility patterns, and secondarily to inflammatory processes [46, 47, 48]. In line with this hypothesis, end‐of‐treatment analyses demonstrated a robust impact of VSL#3‐HS on colonic inflammation. We observed that the stabilized probiotic formulation reduced the production of key interleukins involved in the pathophysiology of IBD (TNF‐α, IL‐1β, and IL‐6), despite unchanged local MPO activity. This apparent dissociation may reflect distinct mechanisms (cytokine suppression and inhibition of enzymatic activity) or time‐dependent effects. In particular, MPO activity is closely linked to antimicrobial properties, as it generates reactive oxygen species to eliminate foreign particles [49]. We hypothesize that bacterial dysbiosis may still have been present at the time of tissue sampling, potentially explaining the persistence of MPO enzyme activity. Analysis of barrier integrity revealed a partial effect of VSL#3^−^HS consumption on the recovery of tight junction (occludin) gene expression, contributing to the observed health benefits. The lack of histological recovery likely reflects the short treatment duration, which was insufficient to induce microscopic changes. In line with this, sustained MPO activity suggests neutrophil infiltration [50], which both supports and amplifies the inflammatory response while contributing to tissue damage. These findings provide insight into mechanisms of probiotic action, which remain incompletely understood. By bypassing the need for bacterial viability, postbiotics ensure rapid effects and enhanced safety in vulnerable populations, including the immunosuppressed. Unlike live probiotics, which carry a minimal risk of systemic infections (e.g., bacteremia) in frail patients [51], heat‐stabilized formulations provide a safer alternative. This expands the reach of biotherapy to broader patient cohorts, including those for whom live probiotics are contraindicated.

While this study provides compelling preliminary evidence for the beneficial effects of the heat‐stabilized probiotic formulation VSL#3‐HS in a murine model of acute colitis, several issues remain to be addressed and will guide future investigations. For instance, the short duration of the treatment may have limited the ability to observe full recovery at the histological level and restoration of enzymatic markers such as MPO activity. Extending the treatment window in future studies could clarify the time‐dependent dynamics of inflammation resolution and tissue regeneration. Second, although the study focused on key inflammatory and barrier integrity markers, a comprehensive analysis of gut microbiota composition and its functional modulation by VSL#3‐HS remains to be conducted. This would provide crucial insights into the underlying mechanisms driving the observed anti‐inflammatory effects.

## 5 Conclusions and Perspectives

Overall, this exploratory study demonstrates for the first time that a multi‐strain heat‐stabilized probiotic ameliorates clinical and molecular parameters of colitis in an acute model, complementing previous findings by Sang LX [21] in a chronic setting and highlighting its potential for clinical translation in IBD.

## Author Contributions

**Santucci Chiara**: methodology, formal analysis, investigation, data curation. **Torcinaro Alessio**: methodology, formal analysis, investigation, data curation. **De Santa Francesca**: conceptualization, formal analysis, investigation, data curation, writing – original draft preparation, writing – review and editing, funding acquisition. **Giustiniani Maria Cristina**: methodology, formal analysis, investigation, data curation, writing – original draft preparation, writing – review and editing. **Mora Diego**: methodology, formal analysis, investigation, data curation, writing – original draft preparation, writing – review and editing. **Arioli Stefania**: methodology, formal analysis, investigation, data curation, writing – original draft preparation, writing – review and editing. **Laterza Lucrezia**: formal analysis, investigation, data curation, writing – original draft preparation, writing – review and editing. **Strimpakos Georgios**: conceptualization, formal analysis, investigation, data curation, writing – original draft preparation, writing – review and editing, funding acquisition. **Farioli‐Vecchioli Stefano**: conceptualization, formal analysis, investigation, data curation, writing – original draft preparation, writing – review and editing, funding acquisition. **Petrella Carla**: conceptualization, formal analysis, investigation, data curation, writing – original draft preparation, writing – review and editing, funding acquisition. All authors have read and agreed to the published version of the manuscript.

## Funding

This study was funded by Beingpharma s.r.l (Italy), which also provided both the VSL#3‐HS product and the placebo. Beingpharma s.r.l had no role in study design, data collection and analysis, decision to publish, or preparation of the manuscript.

## Conflicts of Interest

The authors declare no conflicts of interest. Prof. Diego Mora and Dr. Lucrezia Laterza sit on the Scientific Advisory Board of Actial Farmaceutica Srl, and has provided, as a part of his Institutional Activity, technical reports for this company and Beingpharma Srl.

## Supporting information



The following supporting information can be downloaded: supplementary materials and methods: evaluation of VSL#3‐HS microbiological quality; supplementary results: Table S1: quantification of viable (AFc) and dead cells (nAFc) by flow cytometry in VSL#3‐HS. Table S2: quantification of culturable VSL#3‐HS by standard plating in HHD medium. Table S3: microbial strain taxonomy. Figure S1: daily solution consumption (tap water or DSS solution) during the treatment period. Figure S2: amplification of the region encompassing the 54 indel in gene Balac_0771 of *B. animalis subsp. lactis*. **Supporting File**: mnfr70594‐sup‐0001‐SuppMat.docx.

## Data Availability

The data that support the findings of this study are available from the corresponding author upon reasonable request.
