# Supplementary material for: Protective Effect of a Heat‐Stabilized Bacteria Probiotic Preparation (VSL#3‐HS) in a Mouse Model of Ulcerative Colitis Induced by Dextran Sulfate Sodium
Source: Mol Nutr Food Res. 2026 Aug 21;70(16):e70594. doi: 10.1002/mnfr.70594 (PMC13494568; doi:10.1002/mnfr.70594)
Supplement: Supplementary file 1 [file MNFR-70-e70594-s001.docx]

**SUPPLEMENTARY MATERIALS**

**Non-viable probiotic preparation**

Based on the information provided by the supplier, after the fermentation process to replicate live bacteria, each strain has been individually heat-stabilized and then mixed, according to the same proportion used for VSL#3^®^ products. The optimization process ensured cell stabilization while preserving the overall cell integrity. Heat stabilization parameters (time and temperature) and manufacturing process are a CSL srl trade secret.

**Evaluation of VSL#3**^®^**-HS microbiological quality**

Before administration to mice, the product VSL#3^®^-HS undergo a rigorous quality control based on: a) the quantification of the overall viable and dead cells by single cell analysis carried out by flow cytometry, b) the quantification of culturable VSL#3^®^-HS by standard plating in HHD medium, c) Microbial strain taxonomy, identification of microbial species blended in the product VSL#3^®^-HS was carried out by qPCR using primer sets targeted to the single-copy gene *pyk* coding for pyruvate kinase [1].

a) **Total cells quantification**

Total cells quantification and viability evaluation of the VSL#3^®^-HS product (Table S1) was performed in duplicate according to a modified ISO 19344 IDF 232 procedure (protocol B). To this aim, a suspension of product was obtained by weighting 1 g of product powder in 9 ml of PBS (pH 7.4). The suspension was homogenized by stomacher for 120 s at normal speed and serially diluted in PBS (0.1 ml in 0.9 ml). Staining was performed at 37 °C for 15 minutes by addition of 10 µl of 0.1 mM Syto®24 and 10 µl of 0.2 mM PI to the 1 ml of diluted sample. Cell suspensions were analyzed by FCM using a BD Accuri C6 Plus apparatus (Becton Dickinson, Italy) as previously described [1]. The flow cytometry absolute count was performed using the CountBright^TM^ Absolute Counting Beads (Thermo Fisher Scientific, Italy) as reference. Data were reported as active fluorescent cells (AFc), damaged cells (Dc), and non-active fluorescent cells (nAFc) per g of product.

b) **Quantification of culturable VSL#3^®^-HS by standard plating in HHD medium**

The cell suspension obtained by weighting 1 g of product powder in 9 ml of PBS, as above described was serially diluted in the same buffer and plated on HHD medium [2], incubated at 37 °C in anaerobic condition, in triplicate. Data were reported as CFU/g.

c) **Microbial strain taxonomy**

As quality control, all the seven species blended in the product have been identified using a qPCR assay based on species-specific primer-sets targeted to the single-copy gene *pyk* coding for pyruvate kinase [1]. However, this qPCR assay is not able to discriminate between the two highly related *B. animalis* subsp. *lactis* strains present in the VSL#3^®^-HS blend which is composed of a total of eight strains belonging to seven different species [3]. Therefore, the presence of two different strains of *Bifidobacterium* *animalis* subsp. *lactis* was accessed through the identification and the 54 bp insertion within the gene Balac_0771, which codes for a long-chain acyl-CoA synthetases, in strains BL03 and the relative 54 bp deletion in strain BI04. The 54 bp indel was specifically accessed by a PCR assay carried out on total DNA extracted from VSL#3^®^-HS using the primer set bl04_insdel_fw (GGTTTACAACGCAGCTTCCCAGAA) and bal_insdel_rw (CGTCGTCGATATACCCGAGATCG) targeted to the region encompassing the 54 bp indel. To this aim 100 ng of total DNA was used in a PCR mix containing 0.024 U/µl DreamTaq DNA Polymerase (ThermoFisher Scientific) and dNTPs according to the manufacturer’s instructions. PCR amplification was carried out using a Mastercycler nexus eco (Eppendorf, Milan, Italy) and the following thermal protocol: 95 °C for 3 min followed by 35 cycles, each cycles consisting of 95 °C for 45 s, 68 °C for 45 s and 72 °C for 45 s. PCR amplified fragments were analyzed by agarose gel electrophoresis (2.5 % p/v), stained with ethidium bromide and photographed under UV light.

**SUPPLEMENTARY RESULTS**

**Fig S1** Daily solution consumption (tap water or DSS solution) during the treatment period. Graph illustrates the mean ± S.E.M. of daily solution intake for each experimental group (n = number of mice/group) PLA = n.5, DSS = n. 8, DSS + VSL#3®-HS= n.10: two-way ANOVA followed by Tukey’s multiple comparison.

**Evaluation of VSL#3**^®^**-HS microbiological quality**

Quantification of live and dead cells in VSL#3^®^-HS by flow cytometry revealed no viable cells (< 10^8^ FU/g) and dead cells of 2.2 10^11^ ± 2 10^10^ FU/g (Table S1). No viable or dead cells were detected in the placebo formulation. Quantification of culturable VSL#3^®^-HS by standard plating in HHD medium overcomes the low sensitivity of flow cytometry in detecting live cells in high cell density products such as VSL#3^®^-HS. The data obtained revealed the presence of 3.0 x 10^5^ CFU/g, equivalent to the 0.00015% of culturable VSL#3^®^-HS based on the total cell count of the product obtained by flow cytometry. No culturable bacteria have been detected in the placebo.

**Table S1. Quantification of viable (AFc) and dead cells (nAFc) by flow cytometry in VSL#3^®^-HS.**

| **Sample** | **AFc**  (FU/g) | **nAFc**  (FU/g) | **tot**  (FU/g) |
| --- | --- | --- | --- |
| VSL#3^®^-HS, batch NTL3999 | *< 10 ^8^* | *2.2 x 10^11^ ± 2 x 10^10^* | *2.2 x 10^10^ ± 2 x 10^10^* |
| Placebo, batch NTL4001 | *< 10 ^8^* | *< 10 ^8^* | */* |

Microbial cells density reported as fluorescent units (FU) per g of lyophilized VSL#3^®^-HS powder. All data are reported as the average of two independent measurement ± the standard deviation.

**Table S2**. **Quantification of culturable VSL#3^®^-HS by standard plating in HHD medium.**

| **Sample** | **CFU/g** |
| --- | --- |
| VSL#3^®^-HS, batch NTL3999 | *3.0 x 10^5^ ± 4.0 x 10^4^* |
| Placebo, batch NTL4001 | *< 100* |

Microbial cells count reported as colony forming units (CFU) per g of lyophilized VSL#3^®^-HS powder. All data are reported as the average of three independent measurement ± the standard deviation.

Microbial strain taxonomy, assessed by species-specific qPCR assay, confirmed the presence of all the seven declared species (Table S3). The presence of the two strains of *B. animalis* subsp. *lactis* was assessed by detecting the 54 bp indel within the gene Balac_0771 by a strain-specific PCR assay (Fig S2). The overall data obtained confirmed the declared strains composition of VSL#3^®^-HS.

**Table S3. Microbial strain taxonomy**

| qPCR assay target | qPCR results | *Ct* |
| --- | --- | --- |
| *Bifidobacterium animalis* subsp. *lactis* | positive | 18.25 |
| *B. breve* | positive | 24.81 |
| *Lactobacillus acidophilus* | positive | 19.15 |
| *L. helveticus* | positive | 22.32 |
| *L. paracasei* | positive | 20.15 |
| *L. plantarum* | positive | 22.01 |
| *Streptococcus thermophilus* | positive | 18.55 |

**Fig S2.** Amplification of the region encompassing the 54 indel in gene Balac_0771 of *B. animalis* subsp. *lactis*. DNA extracted from pure culture of strain BL03 and Bi04 (Douillard et al., 2018) was used as reference. Total DNA extracted from VSL#3^®^-HS batch NTL3999 revealed the presence of the 464 bp amplified fragment associated to strain BL03 and the presence of the 518 bp amplified fragment associated to strain Bi04. **M**, molecular weight marker Gene Ruler 1kb DNA ladder (ThermoFisher).

**References:**

[1]	D. Mora, R. Filardi, S. Arioli, S. Boeren, S. Aalvink, and W. M. De Vos, “Development of omics‐based protocols for the microbiological characterization of multi‐strain formulations marketed as probiotics: the case of VSL#3,” *Microb. Biotechnol.*, vol. 12, no. 6, pp. 1371–1386, Nov. 2019, doi: 10.1111/1751-7915.13476.

[2]	L. C. McDonald, R. F. McFeeters, M. A. Daeschel, and H. P. Fleming, “A Differential Medium for the Enumeration of Homofermentative and Heterofermentative Lactic Acid Bacteria,” *Appl. Environ. Microbiol.*, vol. 53, no. 6, pp. 1382–1384, Jun. 1987, doi: 10.1128/aem.53.6.1382-1384.1987.

[3]	F. P. Douillard, D. Mora, R. T. Eijlander, M. Wels, and W. M. De Vos, “Comparative genomic analysis of the multispecies probiotic-marketed product VSL#3,” *PLOS ONE*, vol. 13, no. 2, p. e0192452, Feb. 2018, doi: 10.1371/journal.pone.0192452.
